# Eating behavior patterns, metabolic parameters and circulating oxytocin levels in patients with obesity: an exploratory study

**DOI:** 10.1007/s40519-024-01698-w

**Published:** 2025-01-17

**Authors:** Elena Colonnello, Flavia Libotte, Davide Masi, Mariaignazia Curreli, Chandra Massetti, Orietta Gandini, Elena Gangitano, Mikiko Watanabe, Stefania Mariani, Lucio Gnessi, Carla Lubrano

**Affiliations:** 1 Department of Experimental Medicine, Section of Medical Pathophysiology, Food Science and Endocrinology, Sapienza University of Rome “La Sapienza”, Policlinico Umberto Viale del Policlinico 155 – 00161, Rome, Italy; 2 Chair of Endocrinology and Medical Sexology (ENDOSEX), Department of Systems Medicine, University of Rome Tor Vergata, Rome, Italy; 3 Department of Molecular Medicine, Sapienza University of Rome, Rome, Italy

**Keywords:** Oxytocin, Food addiction, Eating disorder, Obesity, Emotional eating

## Abstract

**Purpose:**

Obesity is a complex heterogeneous disease often associated with dysfunctional eating behavior patterns. Oxytocin (OT) is a neurohormone involved in the regulation of energy metabolism and eating behavior. The aim of the present study was to evaluate in a population of patients with obesity circulating levels of OT and dysfunctional eating behaviors in relation to anthropometric, hormonal and metabolic parameters.

**Methods:**

A prospective, observational, single-center study was conducted at the Center of High Specialization for the Care of Obesity of Sapienza University of Rome. Adult subjects with body mass index (BMI) ≥ 30 kg/m2 were recruited. Body impedance assessment (BIA), biochemical and hormonal parameters, plasma OT concentration analysis and the Eating Behaviors Assessment for Obesity (EBA-O) questionnaire were evaluated.

**Results:**

A total of 21 patients, 16 females and 5 males, with a mean age of 45.7 ± 15.1 years, mean BMI of 40.89 ± 8.02 kg/m^2^ and plasma OT concentration of 1365.61 ± 438.03 pg/mL were recruited. The dysfunctional eating behavior traits investigated by the EBA-O appear significantly associated with metabolic derangements. In particular, night eating is associated with alterations in lipid metabolism (*p* < 0.01). Circulating OT correlates positively with BMI (*r* = 0,43; *p* < 0.05), and Hepatic Steatosis Index (HIS) (*r* = 0.46; *p* < 0.05), while its role in subjects with obesity and alterations in glucose metabolism is less clear. Interestingly, circulating OT levels < 1312.55 pg/mL may be predictive of food addiction (100% sensitivity; 62.5% specificity).

**Conclusions:**

Despite the need for larger studies to confirm their validity, the clinical utility of the EBA-O and circulating OT in identifying dysfunctional eating behaviors appears promising.

**Supplementary Information:**

The online version contains supplementary material available at 10.1007/s40519-024-01698-w.

## Introduction

Obesity is a widespread, complex, progressive, and relapsing chronic disease. It is characterized by abnormal or excessive accumulation of body fat that negatively affects physical and mental health, increases the risk of long-term medical complications, and reduces the length and quality of life [1–5]. In a simplistic view, obesity results from a disequilibrium in the energy balance: an intake of calories that exceeds their consumption results in an accumulation of energy in the form of fat [6–8]. From a more complete pathophysiological perspective, this complex disease has a multifactorial etiology, being caused by the interaction of multiple biological [9–12], behavioral [13–15] and environmental factors [16, 17].

According to the WHO Global Health Observatory, about 16% of adults aged 18 years and older worldwide were obese in 2022, with the overall prevalence of obesity that has more than doubled between 1990 and 2022 [18]. Overweight and obesity are among the leading causes of death and disability in the European region [19]. Obesity also has a large economic impact: in most OECD (Organization for Economic Cooperation and Development) countries it is responsible for about 1–3 percent of total health expenditures [20].

Dysfunctional eating behaviors can affect the pathogenesis and maintenance of excess weight and severely limit the long-term effectiveness of treatment [21–23]. A relevant role is played especially by those related to emotions, as resumed in Table 1.

**Table 1:** Most common dysfunctional eating behaviors encountered in overweight/obese people

Name	Definition	Reference
Binge eating	Eating a large amount of food in short time with the feeling of loss of control over eating	[61]
Grazing	Repetitive consumption (more than twice) of small/moderate amounts of food in an unplanned manner, with or without compulsive characteristics	[62]
Night eating	Recurrent episodes of night eating, as manifested by eating after awakening from sleep or by excessive food consumption after the evening meal that causes significant distress and/or impairment in functioning	[61]
Food addiction	Condition characterized by addiction in relation to some high-fat and high-carbohydrate foods that leads to clinically significant impairment or distress on several areas of functioning	[63]
Sweet eating	Eating behavior in which at least 50% of daily consumed carbohydrates consist of simple carbohydrates and which can be triggered by emotional factors (i.e., stress)	[28]
Emotional eating	Broad and ambiguous concept that involves eating in response to emotionally charged or stressful situations, with loss of control over eating high-calorie meals without being hungry	[64]

There are several instruments that separately assess pathological eating behaviors. The most frequently used in the population with obesity are: *Binge Eating Scale* [24]*, Night Eating Questionnaire* [25]*, Yale Food Addiction Scale 2.0* [26]*, Grazing Questionnaire* [27]*, Dutch Sweet Eating Questionnaire* [28]*.* Nevertheless, often the administration of these tests is time-consuming, and the interpretation may require specialized training. Recently, Segura-Garcia C, et al*.* have developed and validated the EBA-O (*Eating Behaviors Assessment for Obesity*) to assess simultaneously the presence of 5 dysfunctional eating behaviors (food addiction, night eating, binge eating, sweet eating and hyperphagia) [29], which has shown to be an easy-to-use and practical tool even in non-specialized settings [30].

Oxytocin (OT) is a neurohypophyseal hormone known for its functions in childbirth and lactation. It has recently gained attention for its role in energy balance, pro-social behaviors, and other areas pertinent to obesity management [31–33]. It appears to reduce food intake related to hedonic and homeostatic drive by enhancing the activity of brain regions that exert cognitive control [34]. It suppresses the activity of endocrine stress axes [33, 35] and links psychosocial functions and eating behavior [36–38]. In addition to its central action, OT exerts peripheral metabolic effects: it increases glucose tolerance and insulin sensitivity; it enhances net hepatic glucose oxidation; in adipose tissue, OT increases lipolysis and β-oxidation of fatty acids [31].

OT is deemed as a marker of energy availability and a possible mediator of bone density, strength, and structure [32, 39]. In preliminary studies, it also appears to be a predictor of weight loss [40].

Recent studies showed parallel changes in OT levels in plasma and CNS that would suggest common regulatory processes and, therefore, support the use of peripheral OT as a mirror of central OT [41]. However, being present in concentrations 100 million to ten billion times lower (pg/ml vs. g/l) than albumin in human plasma, measuring the concentrations of OX accurately has proven difficult, with a lack of a standardized measurement protocol and a consensus as to the optimum assay conditions that should be used [42]. Moreover, given the short half-life in human plasma, proteases are commonly used to increase its stability, the commonest being the serine protease inhibitor aprotinin [43].

That said, identifying obesity phenotypes—based on eating behavior, metabolic parameters, body composition, and circulating levels of OT—could enable better clinical management of patients and the development of individualized treatment plans [15, 29, 44].

The aim of the study was thus to evaluate in a population of patients with obesity the relationships between circulating OT levels and dysfunctional eating behaviors investigated by EBA-O in relation to clinical, anthropometric, metabolic, and body composition parameters. Given the pandemic spread of obesity [45] and its impact on contemporary society, there is indeed a clear urgency to identify effective strategies for the treatment of this complex and heterogeneous disease.

## Materials and methods

### Study design and patients’ enrollment

A prospective, observational, single-center study was conducted in Center of High Specialization for the Care of Obesity (CASCO), Department of Experimental Medicine, Section of Medical Pathophysiology, Food Science and Endocrinology, University of Rome “La Sapienza”, in Italy.

Patients were enrolled according the following criteria. Inclusion criteria: age between 18 and 65 years; BMI ≥ 30 kg/ m^2^; willingness to sign an informed consent. Exclusion criteria: pregnancy/lactation status; inability to complete the various assessments; drug abuse; neurological diseases (e.g., spinal cord injury, multiple sclerosis, parkinsonism); severe psychiatric comorbidities (e.g., major depression, schizophrenia, bipolar disorder); lack of informed consent.

No gender ratio was set upon enrolment.

### Measurements

#### Clinical-anthropometric evaluations

Anthropometric measurements included weight (kg), height (cm), body mass index (BMI—kg/m2), waist circumference (WC—cm), hip circumference (HCcm), waist-to-hip ratio (WHR), systolic blood pressure (SBP—mmHg), diastolic blood pressure (DBP—mmHg) and heart rate (HR—bpm). Body weight and height were measured between 8:00 and 10:00 in fasting and empty bladder subjects, with light clothing and without shoes. A calibrated balance‐beam (200 kg ± 0.1 kg) and a stadiometer (200 cm ± 0.1 cm) were used for recording weight and height respectively (Seca GmbH & Co). The BMI was calculated as weight in kilograms divided by the square of the height in meters.

WC was measured using a flexible, inelastic tape measure positioned at the anterior superior iliac spine, at the end of normal exhalation. Instead, hip circumference was measured at the level of a horizontal plane passing through the widest point of the hips above the greater trochanter. Waist-to-hip ratio (WHR) was then calculated as WC divided by HC. Blood pressure was measured by GAMMA XXL LF-S 2-tube rail aneroid sphygmomanometer (HEINE Optotechnik, Herrsching) using the dedicated cuff for obesity.

#### Body composition evaluation and visceral adiposity index (VAI)

Body composition was obtained by bioelectrical impedance analysis (BIA), using the machine BodygramPlus 1.2.2.8 © Akern 2016.

The examination was performed with the fasting patient in a supine, relaxed position, arms and legs slightly apart. Four disposable electrodes were placed per subject (tetrapolar examination), two distal injectors (hand, forefoot) and two proximal detectors (wrist, ankle).

The visceral adiposity index (VAI) is an empirical-mathematical model, gender-specific, based on simple anthropometric (BMI and WC) and functional parameters [triglycerides (TG) and HDL cholesterol (HDL)], and indicative of fat distribution and dysfunction and the associated cardio-metabolic risk, with age-stratified cut-off points [46]. VAI was determined depending on gender using the following equations:

$${\text{Females}}:{\text{VAI}} = \, \left[ {{\text{WC }} \div \, \left( {36.58 \, + \, 1.89 \, \times {\text{ BMI}}} \right)} \right] \, \times \, \left( {{\text{TG }} \div \, 0.81} \right) \, \times \, \left( {1.52 \, \div {\text{HDL}}} \right);$$

$${\text{Males}}:{\text{VAI}} = \, \left[ {{\text{WC }} \div \, \left( {39.68 \, + \, 1.88 \, \times {\text{ BMI}}} \right)} \right] \, \times \, \left( {{\text{TG }} \div 1.03} \right) \, \times \, \left( {1.31 \, \div {\text{HDL}}} \right).$$

#### Eating behaviors assessment for obesity (EBA-O)

The questionnaire selected for our study to assess eating behavior is the “Eating Behaviors Assessment for Obesity” (EBA-O). It has proven to be a valid, reliable and practical tool for clinicians, even those without expertise in the field of eating disorders [29].

The test consists of 18 items with multiple-choice answers that provide an overall mean score (total score) and five domains (“factors”) associated with a specific pathologic eating behavior, namely food addiction, night eating, binge eating, sweet eating and hyperphagia.

The cutoff established for each domain and for the EBA-O total score is a value ≥ 4.

After compiling the test, each patient also performed a consultation with a psychologist specialized in obesity and eating disorders, as part of the routine multidisciplinary evaluation.

### Laboratory assay

Peripheral blood samples were collected from participants after an overnight fast of at least 8 h. Biochemical assessment included routine glyco-metabolic panel, thyroid function, estradiol in follicular phase in women and testosterone in males.

The homeostatic model assessment of insulin resistance (HOMA-IR) was calculated using the formula [65]:

$${\text{HOMA}} - {\text{IR }} = {\mkern 1mu} {{\left( {{\text{insulin mU/L}}{\mkern 1mu} \times {\text{ glucose mg/dL}}} \right){\mkern 1mu} } \mathord{\left/ {\vphantom {{\left( {{\text{insulin mU/L}}{\mkern 1mu} \times {\text{ glucose mg/dL}}} \right){\mkern 1mu} } {405^{{51}} .}}} \right. \kern-\nulldelimiterspace} {405}}{\mkern 1mu}$$

The cut-off suggestive for the presence of insulin-resistance is ≥ 2,5.

The hepatic steatosis index (HSI) is a simple screening tool reflecting non-alcoholic fatty liver disease (NAFLD), calculated with the formula:

$$8 \, \times \, \left( {{\text{ALT}}/{\text{AST}}} \right) \, + {\text{ BMI }} + \, \left( {2,{\text{ if diabetes mellitus}}} \right) \, + \, \left( {2,{\text{ if female}}} \right),$$

with values < 30 ruling out and values > 36 ruling in steatosis [47].

Plasma OT concentration was performed using a commercial ELISA kit (Antibodies.com, code A74919, Cambridge, UK). The sample was appropriately prepared by adding 500 KIU of aprotinin (Abcam code ab146286, Cambridge, UK) per 10 ml of EDTA-collected whole blood. Aprotinin was added within the first 5 min of collection, and the sample was centrifuged for 14 min at 1400 rpm immediately after the addition of aprotinin. Once the sample was centrifuged, the plasma was stored at − 80 °C until further analysis.

### Data management and statistical analysis

Data analysis was performed via Statsoft statistical software, version 12.0 (Stat Soft, Inc., Tulsa, Oklahoma). Categorical variables were reported as frequency distribution, while continuous variables were described by mean ± standard deviation (SD) unless otherwise stated.

Pearson's linear correlation coefficient *r* was calculated to investigate linear correlations between quantitative variables. A one-way analysis of variance (ANOVA) was used to compare the studied variables between patients with and without dysfunctional eating behavior. Proportions and categorical variables, especially the correlations between eating behavior dysfunction found at EBA-O, were analyzed with the Chi-square test. Receiver operating characteristic curve (ROC) with area under the curve (AUC) was evaluated to test the predictive value of OT toward any pathological eating behavior. *p* values < 0.05 were considered statistically significant.

### Ethical approval

The study was carried out in accordance with the code of ethics of the World Medical Association for human studies (Declaration of Helsinki, 2001) and Good Clinical Practice (GCP). The research protocol (“SUMOTO”) was approved by the Ethical Committee of Sapienza University of Rome (rif. 5475). All patients were informed about the possible risks and benefits of the proposed interventions and signed an informed consent form to voluntarily participate in this study.

## Results

A total of 21 patients with obesity were enrolled, 16 females and 5 males, with a mean age of 45.67 ± 15.07 years. Mean BMI was 40.89 ± 8.02 kg/m^2^ and plasma OT concentration was 1365.61 ± 438.03 pg/mL, ranging from 900.45 to 2518 pg/mL (Table 2).

**Table 2:** Clinical, biochemical and anthropometric characteristics of the study population (n. 21)

Variables	Mean ± SD
Age (years)	45,67 ± 15,07
BMI (kg/m^2^)	40,89 ± 8,02
Waist circumference (cm)	118,34 ± 17,90
Hip circumference (cm)	126,58 ± 15,18
WHR	0,94 ± 0,111
TC/HDL ratio	3,50 ± 0,76
TG/HDL ratio	2,11 ± 1,26
HR (bpm)	74,05 ± 12,70
SP (mmHg)	131 ± 19,07
DP (mmHg)	82,81 ± 6,43
Total cholesterol (mg/dL)	204,32 ± 42,20
LDL (mg/dL)	120,63 ± 30,00
HDL (mg/dL)	60,30 ± 15,86
Triglycerides (mg/dL)	116,52 ± 58,46
Glycemia (mg/dL)	101,99 ± 34,92
Insulin (mUI/mL)	24,95 ± 22,60
HOMA-IR	7,78 ± 12,95
HbA1c%	5,79 ± 1,54
TSH (µUI/mL)	2,32 ± 1,03
FT4 (ng/dL)	1,11 ± 0,24
FT3 (pg/mL)	3,10 ± 0,77
Estradiol (pg/mL) in females	32,12 ± 22,12
Total testosterone (ng/mL) in males	2,97 ± 1,23
Oxytocin (pg/mL)	1365,61 ± 438,03

BMI, body mass index; WHR (waist–hip ratio), waist-to-hip ratio; TC/HDL ratio, total cholesterol/HDL cholesterol ratio; TG/HDL ratio, triglyceride/HDL cholesterol ratio; HR, heart rate; SP, systolic pressure; DP, diastolic pressure; HOMA-IR, homeostasis model assessment of insulin resistance; Hba1c, glycated hemoglobin; TSH, thyrotropin; FT4, free thyroxine T4; FT3, free triiodothyronine T3

The body composition variables are shown in Table 3.

**Table 3:** Body composition by BIA of the study population (n. 21)

Variables	Mean ± SD
PhA (°)	6,1 ± 0,74
BCMI	12,13 ± 2,17
BMR (kcal)	1687,9 ± 241,21
BCM (kg)	33,18 ± 8,12
FFM (kg)	60,09 ± 11,36
FM (kg)	53,08 ± 17,92
TBW (L)	45,69 ± 9,27
ECW (L)	45,69 ± 9,27

PhA, phase angle; BCMI, body cell mass index; BMR, basal metabolic rate; BCM, body cell mass; FFM, lean mass; FM, fat mass; TBW, total body water; ECW, extracellular water

The analysis of the EBA-O scores suggested the presence of a pathological eating disorder (overall total score (≥ 4) in 3/21 patients. According to the established cutoff value for each subdomain (≥ 4 suggesting an altered eating behavior), 2/21 of patients had night eating, 5/21 food addiction, 6/21 sweet eating, 6/21 hyperphagia, and 8/21 binge eating.

**Fig. 1:**
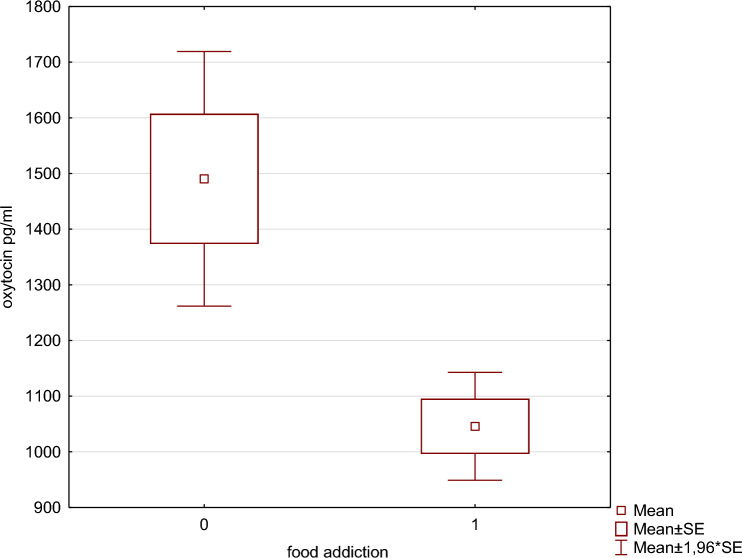
The graphs regarding the association between food addiction and circulating oxytocin levels are shown (*categorized. box & whisker plot*)

One-way ANOVA for the comparison of the studied variables between patients with and without dysfunctional eating behavior, identified by EBA-O in the study population, revealed that (Supplementary Tables 1A;1B,;1C;1D;1E;1F;1G) patients with *night eating* had significantly increased levels of triglycerides (*p* < 0.001), total cholesterol (*p* < 0.05) and TG/HDL ratio (*p* < 0.01);

- subjects who reported *food addiction* had more frequently associated other dysfunctional eating behaviors, such as *sweet eating* (*p* < 0.001), *hyperphagia* (*p* < 0.01), *binge eating* (*p* < 0.01), and a higher *EBA-O total score* (*p* < 0.001); they had a lower waist-to-hip ratio (WHR) (*p* < 0.01) and lower OT values (*p* < 0.01) (Fig. 1);
- patients with *sweet eating* had more frequently associated *hyperphagia* (*p* < 0.05) and a *higher total EBA-O score* (*p* < 0.001);
- people with *hyperphagia* had an increased hepatic steatosis index (HSI) (*p* < 0.05) and a *total EBA-O score* above the cutoff level (*p* < 0.005);
- patients with *binge eating* had a significant increase in *total EBA-O score* (*p* < 0.005);
- having a *Total EBA-O* score above the cutoff level was associated with *night eating* (*p* < 0.05) and lower WHR (*p* < 0.05).

Chi-square test, shows that food addiction is associated with binge eating (*p* < 0.01), sweet eating (*p* < 0.001), and total score (*p* < 0.001) (Supplementary Figs. 1a–c); the presence of sweet eating is accompanied by hyperphagia (*p* < 0.05) (Supplementary Fig. 1d); total EBA-O score positivity with hyperphagia (*p* < 0.001) (Supplementary Fig. 1e).

To investigate the linear correlations between OT levels and the other quantitative variables, Pearson's linear correlation coefficient *r* was calculated. After adjustment for age and sex, plasma OT levels correlated positively with BMI (*r* = 43; *p* < 0.05) (Supplementary Fig. 2) but not with waist circumference or waist-to-hip ratio.

Regarding metabolic assessment, OT correlated positively with HbA1c% (*r* = 0.54; *p* < 0.05) (Supplementary Fig. 3), blood glucose (*r* = 0.51; *p* < 0.01) and HOMA-IR (*r* = 0.55; *p* < 0.01). OT also correlated positively with HSI (*r* = 0.46; *p* < 0.05) (Supplementary Fig. 4) and estradiol (*r* = 0.72; *p* < 0.005) (Supplementary Fig. 5). OT was not significantly correlated with the assessment of lipid parameters, nor to body composition parameters.

Regarding the relationship with dysfunctional eating behaviors, as mentioned above, OT levels were significantly lower in subjects with food addiction (*p* < 0.01) (Fig. 1).

**Fig. 2:**
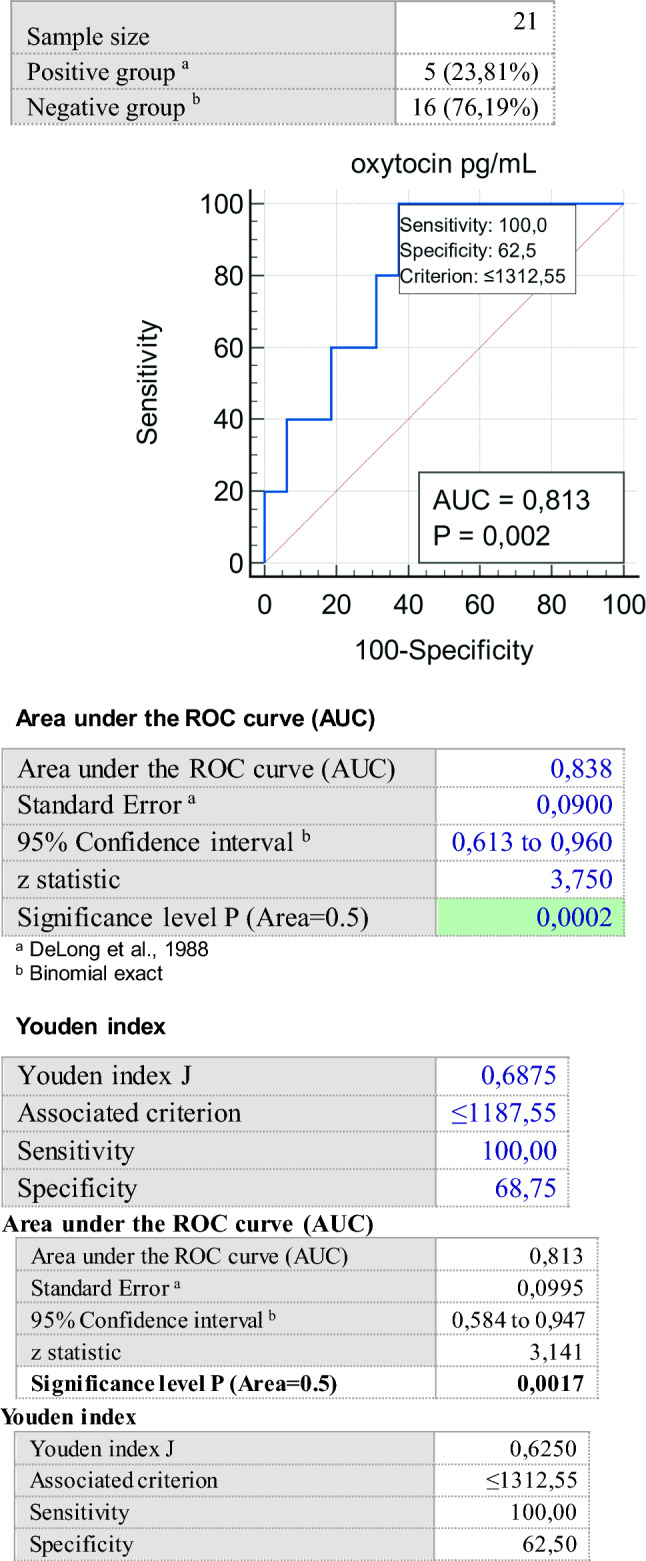
ROC curve of circulating oxytocin (OT) levels and the occurrence of *food addiction*. The predictive outcome of OT is represented as specificity (rate of true positives, x-axis) in relation to sensitivity (rate of false positives, y-axis)

Receiver operating characteristic (ROC) curve of circulating oxytocin (OT) levels versus food addiction domain positivity was constructed: OT levels < 1312,55 pg/ml were predictive of the presence of food addiction with 100% sensitivity and 62,5% specificity (Fig. 2).

## Discussion

In our study, despite the small sample, we observed significant associations between different dysfunctional eating behaviors that are in line with data in the literature. Firstly, subjects with food addiction were also frequently positive on the other domains of the EBA-O (*p* < 0.05), with the exception of night eating. In previous studies, a frequent association between the altered eating behaviors corresponding to the EBA-O domains had been observed; the most common between food addiction and binge eating, the rarest between night eating and hyperphagia [29].

In our study, the presence of food addiction seems also associated with lower plasma OT values (*p* < 0.05). Data in the literature show that differences in oxytocinergic system may influence susceptibility to addiction [48], although the specific relationship between food addiction and OT is still to be clarified. Yet, the correlation between these two variables appears to be direct in some studies, inverse in others [49]. In our study, in the patients whose psychometric assessment suggested the presence of food addiction, it could be speculated that the reduced levels of OT could correspond to a decreased inhibitory action of OT on the endocrine axes of stress, and thus with an increase in pleasurable or compulsive drives, such as that to the consumption of “comfort food” [31].

One interesting result is the association—statistically significant—between night eating positivity and alterations in lipid metabolism. Specifically, this dysfunctional eating behavior correlates with higher levels of total cholesterol, triglycerides, and TG/HDL ratio.

These findings are in line with those in the literature: altered eating behavior patterns, such as night eating, have been associated with an increased probability of having cardiovascular risk factors, such as increased visceral adiposity, obesity, and dyslipidemia [50]. In particular, an association was observed between night eating and coronary heart disease. The mechanisms underlying this could include increased energy intake, a change in appetite, and an alteration of the circadian clock, with a delay not only in the food intake, but in the entire functioning [51, 52]. A large-scale prospective study showed that, in an adult population free of major chronic diseases, habitual night eating is positively associated with the progression of arterial stiffness, a hallmark of atherosclerosis and biological aging [53]. Moreover, NES patients present with elevated eating disorder pathology and higher occurrence of psychotic traits and depressive symptoms than controls [54, 55]. Investigating about the presence of night eating and providing adequate recommendations on correct meal timing—as well as those on nutrients, foods, and meal patterns—may indeed prove useful in these patients, but disordered eating behaviors and emotion dysregulation, as well as poor sleep habits, should also be primarily addressed [54].

In our study, hyperphagia was found to be associated with increased HSI. The dietary pattern of hyperphagia refers to excessive energy intake or overeating during main meals, without loss of control (e.g., larger portions or encores) [56]. The result obtained in our study is coherent with the pathogenetic mechanism underlying NAFLD, a frequent comorbidity of obesity: hypercaloric diet, excess weight, and insulin resistance result in increased free fatty acids and lipogenesis in the liver [4].

A direct correlation has emerged between OT levels and body mass index (BMI). In studies on men, data regarding circulating OT concentrations in subjects with obesity appear discordant: depending on the authors, they are higher, unchanged or decreased, compared with normal-weight [36]. The discrepancy in observations among the various papers could be attributed to differences in the characteristics of enrolled subjects (e.g., with or without metabolic syndrome and diabetes), as well as differences in the assay method used to measure OT [31], in addition to the lack of aprotinin utilization. Although it cannot be considered a definitive opinion, OT has been proposed as a marker of energy availability: the increase in endogenous serum OT observed in women with obesity would signal, in this population, the presence of energy reserves, the need to decrease caloric intake and increase energy expenditure [39]. Studies on the exogenous administration of OT—in rodents with diet-induced obesity and in humans—suggest that subjects with obesity are found to be sensitive to OT and that a further increase in its levels, in obesity, may lead to a compensatory decrease in food intake, adipose tissue stores and/or body weight [33]. Other authors report, in subjects with obesity, a condition of receptor resistance to OT—already detected in patients with anorexia—that could hinder the natural anorectic and weight-loss-inducing effects characteristic of OT [57].

In our sample, only one patient had diabetes mellitus, and OT correlates positively with glycated hemoglobin (HbA1c%) (*r* = 0.56; *p* = 0.02), fasting blood glucose (*r* = 0.51; *p* = 0.017) and HOMA-IR (*r* = 0.55; *p* = 0.014). These data are in opposition to those found in other studies where, in contrast, fasting serum OT is negatively associated with HbA1c%, fasting and postprandial blood glucose, fasting and postprandial insulin, and HOMA-IR [31]. In Qian et al., fasting OT levels in subjects with type 2 diabetes mellitus were then significantly decreased compared with subjects with normal glucose tolerance [53].

OT promotes glucose uptake in skeletal muscle and adipose tissue, stimulates insulin secretion by the pancreas, increases glucose tolerance and insulin sensitivity, and appears to have insulin-like activity on adipose tissue. The apparent discrepancy in our results may suggest that abnormal blood glucose or an oxytocin-resistance state may be modifying factors in the OT-obesity relationship. An intriguing study by Brede et al. revealed that, unlike normal-weight subjects, subjects with obesity do not experience the expected attenuation of glycemic spikes and reactivity to oral glucose load after OT treatment [58].

Since abnormal glycemia or an OT-resistance state may alter the relationship between OT and obesity, this should be considered in further studies to obtain clearer results regarding the OT-BMI relationship as well. For example, patients with both diabetes and obesity should be distinguished from those with obesity who have normal blood glucose, as specified in the study limitations below.

Other positive correlations between OT and biochemical/hormonal parameters were observed with HSI and estradiol in the female population. Given the positive correlation between OT levels and BMI, the association with HSI could be indirectly explained by the fact that the risk of NCDs associated with obesity, such as NAFLD, increases with the increase in BMI [3]. Regarding the correlation with estradiol, this could be explained, in part, by the physiology of the OT system: OT secretion, in addition to being reflexed, is also stimulated by estrogen [33]. Furthermore, in previous studies, the increase in endogenous serum OT in subjects with obesity has been observed mainly in the female population [39].

Our study has several limitations. First, the enrolled sample size is relatively small, given the exploratory nature of the study. Secondly, the interpretability of the data may be affected by the validity of the oxytocin radioimmunoassay instead of the gold-standard mass spectrometry on one hand, and the possible interpretation of the peripheral plasma value oxytocin measurement on the other. Although significant effort was devoted to homogenising the blood measurements, controlling the time of sample collection and processing and storing samples immediately, not every potential confounder can be excluded, such as medications, sexual activity, physical exercise, perceived stress, social interactions, and intra-individual variability [59]. Moreover, the absence of a normal-weight control group could partly reduce the significance of our results. In addition, we did not exclude from our study patients with type 2 diabetes mellitus, who in Qian et al*.* showed lower fasting serum OT levels [60]. Since patients with type 2 diabetes often also have overweight/obesity, the exclusion of patients with undiagnosed diabetes mellitus could further strengthen the positive relationship we report between OT and BMI.

## Conclusions

According to the data we obtained, although in a small sample, dysfunctional eating behavior patterns investigated by EBA-O seem frequently associated with each other and with metabolic derangements; in particular, night eating is associated with alterations in lipid metabolism. Moreover, low circulating levels of OT may be predictive of food addiction; OT correlates positively with BMI, HSI and, in the female population, with follicular estradiol concentrations, while its role in subjects with obesity and alterations in glucose metabolism is less clear.

Dysfunctional eating behaviors are often associated with refractoriness to obesity treatment and weight regain. Identifying obesity phenotypes—based on eating style, metabolic characteristics, and biochemical/hormonal markers, including OT—could better enable patient clinical management and the development of personalized treatment plans. Future clinical studies in a larger population before and after dietary intervention will be needed to confirm the validity of EBA-O and circulating OT in identifying dysfunctional eating behaviors that can influence response to medical therapy.

## Supplementary Information

Below is the link to the electronic supplementary material.

Supplementary file 1 (DOCX 209 kb)

## Data Availability

No datasets were generated or analysed during the current study.
