# Correction: Eating behavior patterns, metabolic parameters and circulating oxytocin levels in patients with obesity: an exploratory study

**DOI:** 10.1007/s40519-025-01744-1

**Published:** 2025-04-05

**Authors:** Elena Colonnello, Flavia Libotte, Davide Masi, Mariaignazia Curreli, Chandra Massetti, Orietta Gandini, Elena Gangitano, Mikiko Watanabe, Stefania Mariani, Lucio Gnessi, Carla Lubrano

**Affiliations:** 1 Department of Experimental Medicine, Section of Medical Pathophysiology, Food Science and Endocrinology, Sapienza University of Rome, “La Sapienza”, Policlinico Umberto Viale del Policlinico 155-00161, Rome, Italy; 2 Chair of Endocrinology and Medical Sexology (ENDOSEX), Department of Systems Medicine, University of Rome Tor Vergata, Rome, Italy; 3 Department of Molecular Medicine, Sapienza University of Rome, Rome, Italy

**Correction: Eating and Weight Disorders - Studies on Anorexia, Bulimia and Obesity (2025) 30:6** 10.1007/s40519-024-01698-w

In the original version of this article [1], the given and family names of all the authors were incorrectly structured. The names were displayed correctly in all versions at the time of publication.

The original article has been corrected.
