# Supplementary material for: Eating behavior patterns, metabolic parameters and circulating oxytocin levels in patients with obesity: an exploratory study
Source: Eat Weight Disord. 2025 Jan 17;30(1):6. doi: 10.1007/s40519-024-01698-w (PMC11742293; doi:10.1007/s40519-024-01698-w)
Supplement: Supplementary file 1 — (DOCX 209 kb) [file 40519_2024_1698_MOESM1_ESM.docx]

1. *Night eating*

| **VARIABLES** | \| **0 (n.19)** \| \| --- \| | \| **1 (n.2)** \| \| --- \| | \| **p** \| \| --- \| |
| --- | --- | --- | --- | --- | --- | --- |
| \| TG (mg/dL) \| \| --- \| | 103,32±43,17 | 242,00±2,83 | <0,001 |
| \| TG/HDL ratio \| \| --- \| | 1,91±1,15 | 4,01±0,38 | <0,01 |
| \| TCOL (mg/dL) \| \| --- \| | 199,00±40,73 | 254,83±10,37 | <0,05 |
| OT (pg/mL) | 1418,42±461,63 | 1163,810±210,35 | n.s. |

1. *Food addiction*

| **VARIABLES** | \| **0 (n.16)** \| \| --- \| | \| **1 (n.5)** \| \| --- \| | \| **p** \| \| --- \| |
| --- | --- | --- | --- | --- | --- | --- |
| \| Total score EBA-O \| \| --- \| | 1,73±1,04 | 4,35±1,21 | <0,001 |
| \| Sweet eating \| \| --- \| | 2,29±1,66 | 5,60±1,85 | <0,001 |
| \| Binge eating \| \| --- \| | 1,98±2,36 | 5,07±1,19 | <0,01 |
| \| Hyperphagia \| \| --- \| | 1,81±1,72 | 4,27±2,23 | <0,01 |
| \| WHR \| \| --- \| | 0,97±0,09 | 0,84±0,09 | <0,01 |
| \| OT (pg/mL) \| \| --- \| | 1452,99±463,09 | 1086,02±164,00 | <0,01 |

1. *Sweet eating*

| **VARIABLES** | \| **0 (n.15)** \| \| --- \| | \| **1 (n.6)** \| \| --- \| | \| **p** \| \| --- \| |
| --- | --- | --- | --- | --- | --- | --- |
| \| Total score EBA-O \| \| --- \| | 1,71±1,01 | 3,97±1,55 | <0,001 |
| \| Hyperphagia \| \| --- \| | 1,82±1,52 | 3,83±2,75 | <0,05 |
| \| OT (pg/mL) \| \| --- \| | 1401,57±441,35 | 1375,67±503,46 | n.s. |

1. *Hyperphagia*

| **VARIABLES** | \| **0 (n.15)** \| \| --- \| | \| **1 (n.6)** \| \| --- \| | \| **p** \| \| --- \| |
| --- | --- | --- | --- | --- | --- | --- |
| \| Total score EBA-O \| \| --- \| | 1,78±1,08 | 3,80±1,70 | <0,005 |
| \| HSI \| \| --- \| | 48,52±6,89 | 56,79±12,34 | <0,05 |
| \| OT (pg/mL) \| \| --- \| | 1384,28±400,71 | 1418,90±590,64 | n.s. |

1. *Binge eating*

| **VARIABLES** | \| **0 (n.13)** \| \| --- \| | **1 (n.8)** | **p** |
| --- | --- | --- | --- | --- |
| \| Total score EBA-O \| \| --- \| | 1,60±1,00 | 3,58±2,00 | <0,001 |
| \| OT (pg/mL) \| \| --- \| | 1441,79±508,92 | 1316,79±340,56 | n.s. |

1. *Total score EBA-O*

| **VARIABLES** | \| **0 (n.18)** \| \| --- \| | \| **1 (n.3)** \| \| --- \| | \| **p** \| \| --- \| |
| --- | --- | --- | --- | --- | --- | --- |
| \| Night eating \| \| --- \| | 0,79±1,00 | 2,33±2,00 | <0,05 |
| \| WHR \| \| --- \| | 0,95±0,00 | 0,84±0,00 | <0,05 |
| \| OT (pg/mL) \| \| --- \| | 1437,62±464,65 | 1133,52±211,30 | n.s. |

**Supplementary Tables 1A;1B;1C;1D;1E;1F** Comparison of studied variables between patients with and without dysfunctional eating behavior identified by EBA-O in the study population (One-Way ANOVA). Values are expressed as mean ± standard deviation. 0= negative test; 1= positive test.
Abbreviations: TG, Triglycerides; TSH, Thyrotropin; TG/HDL ratio, Triglyceride/HDL cholesterol ratio; TCOL, Total Cholesterol; EBA-O*,* Eating Behaviour Assessment for Obesity; WHR, Waist-Hip ratio; HSI, Hepatic Steatosis Index.

1. *Food addiction-binge eating*


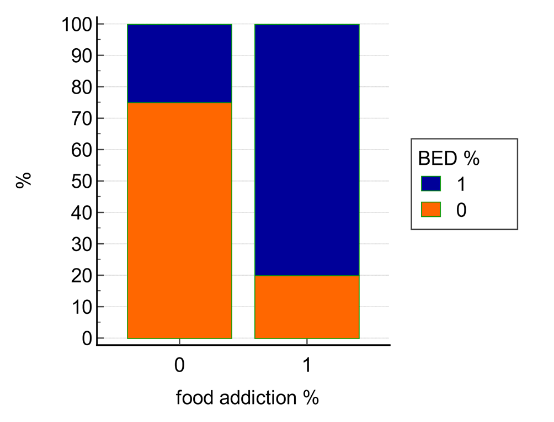


|  | ***Food_addiction*** | |  |
| --- | --- | --- | --- |
| ***Binge eating*** | 0 | 1 |  |
| 0 | 12 | 1 | 13 (61,9%) |
| 1 | 4 | 4 | 8 (38,1%) |
|  | 16 (76,2%) | 5 (23,8%) | 21 |

| Chi-square | 4,654 |
| --- | --- |
| DF | 1 |
| **Significance level** | **P < 0,05** |
| Contingency coefficient | 0,426 |

1. *Food addiction-sweet eating*


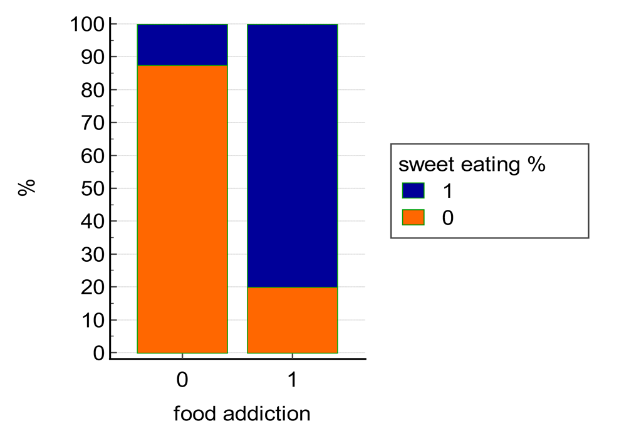


|  | ***Food_addiction*** | |  |
| --- | --- | --- | --- |
| ***Sweet eating*** | 0 | 1 |  |
| 0 | 14 | 1 | 15 (71,4%) |
| 1 | 2 | 4 | 6 (28,6%) |
|  | 16 (76,2%) | 5 (23,8%) | 21 |

| Chi-square | 8,100 |
| --- | --- |
| DF | 1 |
| **Significance level** | **P < 0,005** |
| Contingency coefficient | 0,528 |

1. *Food addiction-total score*


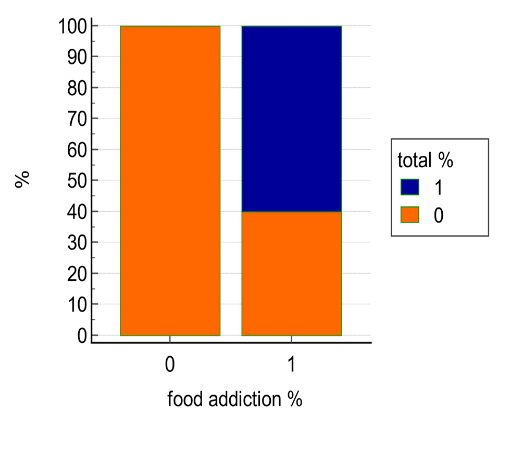


|  | ***Food_addiction*** | |  |
| --- | --- | --- | --- |
| ***Total score*** | 0 | 1 |  |
| 0 | 16 | 2 | 18 (85,7%) |
| 1 | 0 | 3 | 3 (14,3%) |
|  | 16 (76,2%) | 5 (23,8%) | 21 |

| Chi-square | 10,667 |
| --- | --- |
| DF | 1 |
| **Significance level** | **P < 0,001** |
| Contingency coefficient | 0,580 |

1. *Sweet eating- hyperphagia*


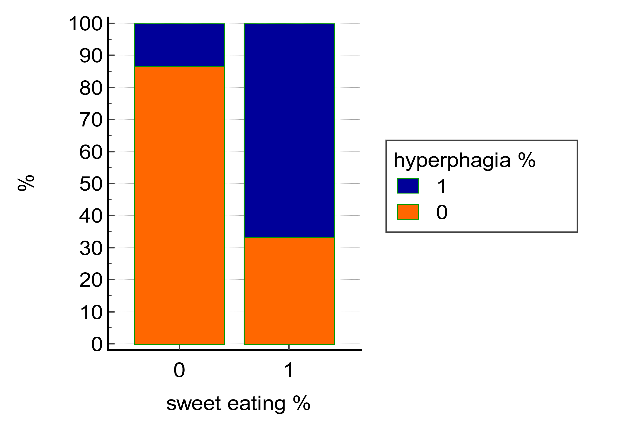


|  | ***Sweet eating*** | |  |
| --- | --- | --- | --- |
| ***Hyperphagia*** | 0 | 1 |  |
| 0 | 13 | 2 | 15 (71,4%) |
| 1 | 2 | 4 | 6 (28,6%) |
|  | 15 (71,4%) | 6 (28,6%) | 21 |

| Chi-square | 5,689 |
| --- | --- |
| DF | 1 |
| **Significance level** | **P < 0,01** |
| Contingency coefficient | 0,462 |

1. *Total score-hyperphagia*


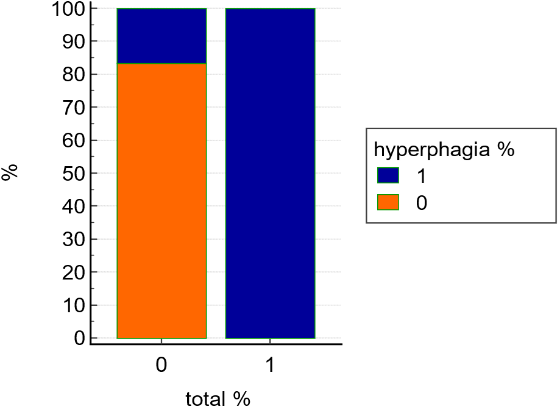


|  | ***Total score*** | |  |
| --- | --- | --- | --- |
| ***Hyperphagia*** | 0 | 1 |  |
| 0 | 15 | 0 | 15 (71,4%) |
| 1 | 3 | 3 | 6 (28,6%) |
|  | 18 (85,7%) | 3 (14,3%) | 21 |

| Chi-square | 8,333 |
| --- | --- |
| DF | 1 |
| **Significance level** | **P < 0,005** |
| Contingency coefficient | 0,533 |

**Supplementary Figures 1 A-B-C-D-E** Chi-square test for correlation analysis between eating behavior disorders found at EBA-O in the study population (n.21). 0= negative test; 1= positive test.

**Supplementary Figure 2.** Direct linear correlation between OT levels and body mass index (BMI) in the study population (n.21).

**Supplementary Figure 3.** Direct linear correlation between OT levels and glycated hemoglobin (HbA1c) in the study population (n.21).

**Supplementary Figure 4.** Direct linear correlation between OT levels and hepatic steatosis index (HSI) in the study population (n.21).

**Supplementary Figure 5.** Direct linear correlation between OT levels and estradiol in the female study population (follicular phase) (n.16).
